# The Interaction Between Dietary Valine and Tryptophan Content and Their Effect on the Performance of Piglets

**DOI:** 10.3390/ani2010076

**Published:** 2012-02-22

**Authors:** Sam Millet

**Affiliations:** Animal Sciences Unit, Institute for Agricultural and Fisheries Research (ILVO), Scheldeweg 68, 9090 Melle, Belgium; E-Mail: sam.millet@ilvo.vlaanderen.be; Tel.: +32-9-272-2615; Fax: +32-9-272-2601

**Keywords:** feed intake, performance, piglet, tryptophan, valine

## Abstract

**Simple Summary:**

To optimize animal performance, pig diets need the right amount of all essential amino acids. Usually, the ideal amino acid profile is used: If the dietary concentration of one particular amino acid is too low, other amino acids will not be used efficiently. In this experiment it was shown that amino acids that stimulate the feed intake may improve the performance of pigs, even if other amino acids are not at the optimal level.

**Abstract:**

Four experimental diets for newly weaned pigs were formulated: (1) low valine and low tryptophan; (2) low valine and high tryptophan; (3) high valine and low tryptophan and (4) high valine and high tryptophan. Dietary standardized ileal digestible (SID) lysine content was 1.06 g/kg. The SID valine to SID lysine ratio was 0.58 and 0.67 for the low and high valine diets, respectively, and SID tryptophan to SID lysine ratios were 0.19 and 0.22 for the low and high tryptophan diets, respectively. In total, 64 pens of 6 pigs (3 barrows and 3 gilts) were divided over the four experimental treatments. No interaction between dietary supply of valine and tryptophan was observed (*P* > 0.1 for all parameters). Increasing the dietary valine content increased the daily feed intake, daily gain and gain:feed (*P* < 0.001 for all three parameters). Increasing the dietary tryptophan content improved gain:feed during the first 2 weeks (*P* < 0.05) and overall (*P* < 0.05). Valine supply had a greater effect on performance results than tryptophan supply. It may thus be beneficial to provide a diet with an optimal dietary concentration of valine even if other amino acids are at suboptimal dietary levels.

## 1. Introduction

The nutrient content of a diet may affect the voluntary feed intake of piglets. Feed intake depends largely on energy density [1,2], so that the daily energy intake remains relatively constant across diets with different energy densities, as long as the density is not too low. On the other hand, feed intake is depressed both by a severe deficiency in the limiting amino acid or an excessive supply of some essential amino acids [2].

When pigs consume energy above maintenance, this will be used for protein and fat deposition [3]. With increasing energy intake, the protein deposition will increase until a maximum (PDmax). Once energy intake leads to PDmax, this protein deposition will stay at that absolute maximum and a further increase in energy intake will not affect protein deposition [4]. In young pigs, feed intake capacity appears to be the main factor limiting protein deposition [4]. Still, increased energy intake will only lead to protein deposition if the composing amino acids are not limiting. While some amino acids can be synthesized by the pigs, other amino acids need to be supplied by the diet. Protein synthesis requires that all necessary amino acids are available to the tissues [5]. For this, the dietary intake of essential and non-essential amino acids needs to meet the requirements. The ideal protein concept is commonly used in feed formulation for pig diets. This concept is defined as the perfect ratio among individual essential amino acids and N required for optimal performance [6]. Amino acid requirements are determined by both the need for maintenance and for protein accretion [6]. It is assumed that, with a sufficient supply of energy and nitrogen, increasing the dietary concentration of a deficient amino acid will only improve performance if the dietary concentration of no other amino acid is limiting growth. If one or more amino acid limits growth, other essential amino acids that are in surplus of the ideal pattern will be catabolized and used as sources of energy and nitrogen for non-essential amino acids [5]. In practice, amino acids are expressed relative to lysine.

Feed conversion is affected by the growth rate and the composition of this growth [7]. Since lean is composed of protein plus water and some lipids, its energetic costs are lower than the costs for fat deposition [8]. With a faster growth rate, the energetic cost for maintenance needs per kg of growth will be lower. Thus, faster growing animals will have a higher feed efficiency and better performance, as long as this faster growth does not lead to fatter animals.

Tryptophan supplementation to a diet that was limiting in tryptophan has been shown to increase piglets’ feed intake [9,10,11]. Also, in studies on valine requirements, suboptimal levels were associated with a lower feed intake [12,13]. Therefore, the hypothesis was tested that adding either valine or tryptophan to a diet short in both valine and tryptophan will lead to an increased feed intake and improved performance.

## 2. Experimental Section

### 2.1. Experimental Design

In 4 batches, 96 pigs (Piétrain boar × hybrid sow) were divided over 16 pens of 6 pigs (3 barrows and 3 gilts per pen) at weaning. Half of the experiment (2 batches) was conducted in spring and the other half in autumn. Each pen was randomly assigned to one of four treatments, with a total of 16 pens per treatment. One pen measured 1 m × 1.8 m (1.8 m²), with 0.3 m² per piglet. The slatted floor was covered with a synthetic coating. The temperature in the compartments ranged from 26 °C at the start to 22 °C at the end of the experiments; the light schedule was natural daylight and supplemental artificial light between 7 h 30 and 15 h 30. The pigs had free access to water and were fed *ad libitum.* The pigs received a standard feed (11.5 g lys/kg, 9.8 MJ net energy per kg) the first week after weaning (at 4 weeks of age) and the experimental feeds between 5 and 9 weeks of age. Four experimental diets were formulated: (1) low valine and low tryptophan (LL); (2) low valine and high tryptophan (LH); (3) high valine and low tryptophan (HL); (4) high valine and high tryptophan (HH). The standardized ileal digestible (SID) lysine concentration was 1.06 g/kg. The dietary lysine concentration was slightly limiting according to the nutrient requirements of pigs from this genotype [14], so that lysine would be limiting and the other amino acids could be formulated relative to lysine. Dietary SID methionine + cystine, threonine, isoleucine, and leucine contents expressed as a percentage of SID lysine were 57, 62, 51, and 96%, respectively (Table 1 and Table 2). The intended SID tryptophan: lysine ratio and SID valine: lysine ratio were 22 and 70, respectively. These were based on optima determined during ILVO research concerning dietary valine concentration (unpublished results) and recent literature on dietary tryptophan content [8]. The intended degree of limitation in the low tryptophan and valine diets were 15% for both amino acids. For valine, a preliminary test at our institute (unpublished data) showed that this degree of restriction would lead to a measurable difference in performance results. For tryptophan, a similar degree of restriction was taken. First, the LL diet was produced and then free L-valine or L-tryptophan were added to yield the intended amount for the different experimental diets. Based on amino acid analysis (Table 2), the eventual ratio of SID tryptophan to SID lysine was 19% in the low tryptophan diets and 22% in the high tryptophan diets. The ratio of SID valine to SID lysine was 58% in the low valine diets and 67% in the high valine diets.

### 2.2. Measurements

All diets were subject to proximate analysis [15,16,17,18]. Analysis of amino acids in the diets was performed by Ajinomoto Eurolysine SAS using a JLC-500/V AminoTac AA Analyzer (Jeol, Croissy-sur-Seine, France) after hydrolysis with 6 N HCl at 110 °C for 23 h under reflux. The Met and Cys contents in diets were determined after performic oxidation before hydrolysis. The Trp content in diets was determined after hydrolysis at 120 °C for 16 h with barium hydroxide and separation by reverse-phase HPLC and fluorometric detection.

All pigs were weighed individually three times: at the beginning of the experiment, 2 weeks into the experiment, and at the end. Average daily gain was calculated as weight gain divided by the number of days. For further analysis, the average per pen was used. Feed consumption per pen was recorded and average daily feed intake was calculated per pen for each period. Feed efficiency per pen was calculated as the total amount of feed consumed by the pen divided by the total gain of all pigs in the pen.

### 2.3. Statistical Analysis

Performance results were analyzed with variance analysis (Statistica 9.0, Statsoft, Tulsa, OK, USA), with tryptophan content, valine content and their interaction as fixed factors and batch as random factor. Results were considered significant if *P* < 0.05. The pen was the experimental unit.

**Table 1:** Ingredient composition of experimental diets (%, as-fed basis).

Item	Low Valine			High Valine	
	Low tryptophan	High tryptophan		Low tryptophan	High tryptophan
Wheat	25.00	24.99		24.97	24.96
Barley	19.76	19.75		19.74	19.73
Corn	18.00	17.99		17.98	17.97
Full fat soybeans	16.00	15.99		15.98	15.98
Soybean meal	5.76	5.76		5.75	5.75
Rapeseed meal	1.91	1.91		1.91	1.91
Beet molasses	4.00	4.00		4.00	3.99
Soy oil	0.20	0.20		0.20	0.20
Mono calcium phosphate	0.78	0.78		0.78	0.78
Salt	0.36	0.36		0.36	0.36
Limestone	0.30	0.30		0.30	0.30
Premix^1^	6.00	6.00		5.99	5.99
Nutrisure^2^	1.00	1.00		1.00	1.00
L-Lysine HCL, 78%	0.49	0.49		0.49	0.49
Dl-methionine, 99%	0.21	0.21		0.21	0.21
L-threonine, 98%	0.20	0.20		0.20	0.20
L-tryptophan, 98%	0.03	0.07		0.03	0.07
L-Valine, 96.5%	–	–		0.11	0.11
Phytase (5,000 IU/g)	0.01	0.01		0.01	0.01

^1^ The premix contained 80% dairy products and 20% vitamin and mineral premix, providing the following quantities of vitamins and minerals per kilogram of basal diet: vitamin A, 15,000 IU; vitamin D3, 2,000 IU; vitamin E, 100 mg; vitamin K, 2 mg; vitamin B1, 2.5 mg; vitamin B2, 7.5 mg; vitamin B5, 20 mg; vitamin B6, 5 mg; vitamin B12, 0.04 mg; vitamin C, 100 mg; vitamin PP, 30 mg; choline, 324 mg; folic acid, 3 mg; biotine, 0.15 mg; Ca, 516 mg; P, 419 mg; Mg, 165 mg; Na, 353 mg; Cl, 1,375 mg; K, 1,227 mg; S, 234 mg; Fe, 100 mg; Cu, 160 mg; Mn, 60 mg; Zn, 100 mg; I, 2 mg; Se, 0.4 mg. ^2^ DSM nutritonal products: A mixture of calcium salts of the following organic acids: Lactic acid, Formic acid, Citric acid monohydrate, Orthophosphoric acid, Propionic acid.

**Table 2:** Analyzed and calculated nutritional composition of the basal diet (as-fed basis) ^*^.

Net energy, MJ/kg ^1^	9.8
Dry matter, %	89.77
Crude ash, %	5.01
Crude fibre, %	3.32
Crude protein, %	17.50
Crude fat, %	4.97
Starches, %	35.09
Sugars, %	8.62
Lactose, %	3.35
ADF, %	4.47
NDF, %	10.81
ADL, %	0.93
Ca,%	0.60
P, %	0.60
Digestible P, %	0.40
Cu, mg/kg	165
Lysine, %	1.19
Methionine + cysteine, %	0.68
Methionine, %	0.43
Threonine, %	0.76
Tryptophan, %	0.24
Isoleucine, %	0.65
Leucine, %	1.22
Valine, %	0.75
Arginine, %	0.95
Histidine, %	0.39
Phenylalanine, %	0.77
SID LYS^1,2^	1.06
SID MET^1,2^	0.39
SID M+C^1,2^	0.60
SID THR^1,2^	0.65
SID TRP^1,2^	0.20
SID ILE^1,2^	0.55
SID LEU^1,2^	1.02
SID VAL^1,2^	0.62

^*^ This is the low valine and low tryptophan diet. Other diets were produced by adding individual amino acids to this diet. ^1^ Values were calculated according to CVB, 2007 [19]. ^2^ SID = Standardized ileal digestible, LYS = lysine, Met= methioninse, M+C = methionine + cysteine, THR = threonine, TRP = tryptophan, ILE= isoleucine, LEU = leucine, VAL = valine.

## 3. Results and Discussion

It has been shown that amino acids may affect feed intake. This increased feed intake will lead to an extra amount of amino acids available for protein deposition, even though they are not perfectly balanced. Therefore, the hypothesis was tested that adding either valine or tryptophan to a diet short in both valine and tryptophan will lead to increased feed intake and improved performance.

No interactions between dietary valine and tryptophan content could be observed (*P* > 0.05 for all parameters, Table 3). However, while valine had a strong effect on feed intake and consequently daily gain (*P* < 0.001 for all parameters), an effect of tryptophan content on daily feed intake or daily gain was not observed (*P* > 0.05).

**Table 3:** Influence of valine and tryptophan concentration on performances of the piglets ^1,2^.

	Low Valine			High Valine		SEM	P		
	Low Tryptophan	High Tryptophan		Low Tryptophan	High Tryptophan		SID VAL	SID TRP	SID VAL x SID TRP
Bodyweight, kg									
5 weeks of age	9.45	9.48		9.40	9.44	0.04	0.514	0.616	0.993
7 weeks of age	14.09	14.41		15.29	15.35	0.12	<0.001	0.185	0.374
9 weeks of age	20.88	21.47		23.51	23.58	0.21	<0.001	0.157	0.267
Daily feed intake, g									
5–7 weeks	565	571		648	636	9	<0.001	0.776	0.430
7–9 weeks	897	899		1004	1008	13	<0.001	0.871	0.952
5–9 weeks	731	735		826	822	11	<0.001	0.871	0.952
Daily weight gain, g									
5–7 weeks	332	352		421	423	60	<0.001	0.194	0.287
7–9 weeks	485	504		587	588	8	<0.001	0.326	0.382
5–9 weeks	408	428		504	505	7	<0.001	0.136	0.215
Feed efficiency, g/g									
5–7 weeks	0.587	0.619		0.652	0.665	0.007	<0.001	0.034	0.377
7–9 weeks	0.544	0.563		0.585	0.584	0.005	0.001	0.313	0.266
5–9 weeks	0.560	0.585		0.610	0.616	0.004	<0.001	0.031	0.159

^1^ Data are means of 16 pens per treatment. ^2^ SID = standardized ileal digestible, VAL= valine, TRP = tryptopan.

The pigs consuming the low valine diets showed a lower feed intake and daily gain. While this has also been observed in some other studies [9,10,20,21], no clear reason has been given. Recent research [22] on mice showed that somatostatin may play a role in anorexia induced by a valine-deficient diet. Central administration of ghrelin and neuropeptide Y restored feed intake in rats on a valine-deficient diet [23]. Systemic administration of ghrelin did not affect the feed intake in these rats [23].

For tryptophan, the stimulating effect on feed intake has been reported [9,10,11]. Tryptophan competes with large neutral amino acids (LNAA) such as leucine, isoleucine and valine for its passage through the blood-brain barrier by sharing a common transport system [24]. Therefore, the ratio of tryptophan to LNAA affects the feed intake [9]. Henry *et al.* [9] assumed that the negative effect of LNAA on feed intake could be explained by a decrease in the tryptophan and consequently serotonin concentration in the brain. However, it cannot be excluded that the assumed positive effect of tryptophan on feed intake is mediated by an inhibition of the L-Leucine uptake to the brain and consequently diminished mTOR-signaling. L-Leucine depresses feed intake through a stimulation of mTOR-signaling [25]. This might explain why an effect of dietary tryptophan on feed intake was not obvious in the present experiment, despite being clear in the experiments of Jansman *et al.* [11] and Henry *et al.* [9]. In their experiments, the leucine: lysine ratio was above 1.5, while in the present experiment, this was around 1. In addition to this, in the study of Henry *et al.* [9], it is possible that the effect they observed was solely caused by the tryptophan:leucine ratio instead of the tryptophan:LNAA concentration, as the leucine concentration varied together with the LNAA concentration.

Dietary tryptophan content did affect the feed efficiency between 5 and 7 weeks of age and between 5 and 9 weeks of age (*P* < 0.05). Also, between 5 and 7 weeks as well as between 7 and 9 weeks of age, the feed efficiency was worse in the groups receiving the low valine diets (*P* ≤ 0.001 for all parameters).  This shows that the LL diet was indeed limiting for both tryptophan and valine.

Despite the absence of an effect of tryptophan on feed intake, it was possible to confirm the hypothesis that the addition of either valine or tryptophan to a diet limiting in both valine and tryptophan may improve performance, even if the other amino acid was limiting growth. Indeed, feed efficiency was improved either by adding tryptophan or by adding valine to the LL diet.

Feed intake may have a role in this. When considering the ideal protein concept, one can assume that the first limiting amino acid determines the amount of protein deposition when sufficient energy is available. The excess amino acids will be catabolized. Therefore, one would expect that only one diet (either HL or LH) would be able to elicit positive growth performance, until the level that the second amino acid becomes limiting. With addition of this second limiting amino acid (the HH diet), further improvement can be expected. However, as valine stimulated the uptake of an extra amount of feed, this provided extra amino acids (including tryptophan) that could be retained as protein tissue. Therefore, it is logical to assume that the energy needs for maintenance were relatively smaller per kg of body weight gain and the feed efficiency improved in the HL *versus* the LL diet, although tryptophan was still limiting maximal protein deposition.

The effect of valine addition to the diet on performance was more pronounced than the effect of tryptophan addition. The levels for the HH feed were based on optima determined during ILVO research concerning dietary valine concentration (unpublished results) and recent literature on dietary tryptophan content [11]. However, the analyzed valine concentration of the basal diet was lower than originally intended, and therefore, the high valine diets might still have been slightly limiting in valine (SID valine: SID lysine was 0.67 instead of 0.70 in the HH diet). This may explain why the 16% increase in valine concentration had a larger effect than the 16% increase in tryptophan concentration. A clear effect of tryptophan addition on feed efficiency was seen in the first 2 weeks of the experiment, but not in the following 2 weeks. It is possible that the pigs responded to the lower tryptophan content through an adaptation mechanism. This may be an improved absorption or a lower catabolism rate of the amino acids. It has been shown that pigs use amino acids more efficiently during a period of amino acid deficiency [26].

The results of this experiment have a direct practical consequence.  Even if other amino acids are formulated to yield suboptimal performance (e.g., because of the limited benefit in comparison to the cost), it may be beneficial to provide the diet with an optimal amount of valine to improve feed intake and performance.

## 4. Conclusions 

It can be concluded that either supplementing valine or tryptophan to a diet that was limiting in both amino acids improved performance of newly weaned piglets, without an interaction between the dietary supply of valine and tryptophan. Valine supply had a greater effect on performance results than tryptophan supply; providing the diet with an optimal amount of valine even if other amino acids are at suboptimal dietary levels may improve performance.
